# Clustering Ensemble Model Based on Self-Organizing Map Network

**DOI:** 10.1155/2020/2971565

**Published:** 2020-08-25

**Authors:** Wenqi Hua, Lingfei Mo

**Affiliations:** School of Instrument Science and Engineering, Southeast University, Nanjing 210096, China

## Abstract

This paper proposes a clustering ensemble method that introduces cascade structure into the self-organizing map (SOM) to solve the problem of the poor performance of a single clusterer. Cascaded SOM is an extension of classical SOM combined with the cascaded structure. The method combines the outputs of multiple SOM networks in a cascaded manner using them as an input to another SOM network. It also utilizes the characteristic of high-dimensional data insensitivity to changes in the values of a small number of dimensions to achieve the effect of ignoring part of the SOM network error output. Since the initial parameters of the SOM network and the sample training order are randomly generated, the model does not need to provide different training samples for each SOM network to generate a differentiated SOM clusterer. After testing on several classical datasets, the experimental results show that the model can effectively improve the accuracy of pattern recognition by 4%∼10%.

## 1. Introduction

Advances in network techniques provide more access to data, especially unlabeled data, which is a significant factor for the fast development of the clustering algorithm. Nowadays, clustering has an important role in areas like pattern recognition, image processing, recommendation system, and data mining. Also, the rising of ensemble learning naturally imports ensemble learning into the clustering, which is called clustering ensemble [1]. It is able, for the clustering ensemble model, to combine results of multiple clusterers while enhancing the performance compared with that of a single clusterer. Meanwhile, the clustering ensemble is robust facing noise or isolated points in input space.

A clustering ensemble model called cascaded self-organizing map (cascaded SOM) is proposed in this paper. Cascaded SOM realizes the ensemble function by learning responses of different clusterers to make a final decision. When learning the responses, the model is learning high-dimensional data, which is insensitive to the change of values in a few dimensions. The characteristic of high-dimensional data makes the model able to ignore the error of several clusterers and output the correct result.

Self-organizing map (SOM) was proposed by Kohonen in 1990 [2]. SOM is a competitive network that consists of an input layer and an output layer (competitive layer). SOM represents the distribution characteristics of input samples on the competitive layer using topographically ordered nodes and, in doing so, achieves clustering through dimensionality reduction. Due to its intrinsic nonlinear mapping capabilities on its low-dimensional neural surface, SOM is distinguished from other widely used clustering algorithms. Such advantage makes SOM a tool of visualizing nonlinear relations of data, topology-based cluster analysis, vector quantization, and projection of multidimensional data. Due to its versatility, SOM has been applied in areas like pattern recognition, image-text processing, data mining, genomics, and medical diagnostics.

Since SOM was proposed, plenty of researchers have put effort to improve it and proposed several variant algorithms. Growing-SOM [3] improves the performance of SOM in incremental learning by replacing the original competitive layer with a variable-scale one; TS-SOM [4] changes the competitive layer from single layer into multiple layers with tree structure, whose recursive characteristic can help to reduce training time; G-SOM [5] introduces genetic algorithm into the parameter initialization step of SOM to generate better parameter, compared with the original randomized parameter initialization method; similar to G-SOM, particle swarm optimization (PSO) can also be applied in the parameter initialization step [6]. Moreover, there is research that compares SOM with an uncommon grid structure and a common grid structure, proving that SOM with uncommon grid structure performs better with data having special distribution [7]. During the past decade, researchers came out with different methods to improve SOM. For example, DASOM introduces denoising autoencoder to reduce the noise in input space [8]; constrained SOM preserves the topology structure by blocking the input space [9]; robust SSGSOM introduces HQ method into semisupervised growing-SOM to improve network robustness [10]. PLSOM uses adaptive learning rate instead of learning rate reduction through training, which can focus on new patterns [11]. PLSOM2 solves the problem that the large error at the early period will lead to a small learning rate in the later period [12], as well as reducing the influence of the noise. Inspired by PLSOM, researchers use the eigenvalue of the autocorrelation matrix of the input vector to control the learning rate, reaching a faster converging speed [13]. In classic SOM, there might be several useless neurons between different patterns, reducing competitive layer utilization. A segmentation method based on the distribution of neurons is proposed to raise the competitive layer utilization of 1D SOM and get a more stable network structure [14].

Each neuron in the SOM competitive layer represents a prototype of one pattern in input space which is more representative than any input that inspires this neuron. Such characteristic is applied in several types of researches. Facing situation where samples of one pattern are far less than those of other patterns, SOM oversampling (SOMO) uses SOM to train prototype of minority samples as the input of SMOTE algorithm, providing oversampling for minority samples as well as avoiding the oversampled data being representative [15]. Also, in the real world, there should be neither samples nor prototypes in certain areas in the input space. Classic SOM is not able to handle this problem. Forbidden region SOM (FRSOM) set forbidden regions according to prior knowledge [16]. During training, FRSOM considers the distance differently; that is, the path which represents the distance between two points must not go across the forbidden area. This strategy ensures that no prototype will be set in the forbidden region, improving the clustering result.

**Figure 1:**
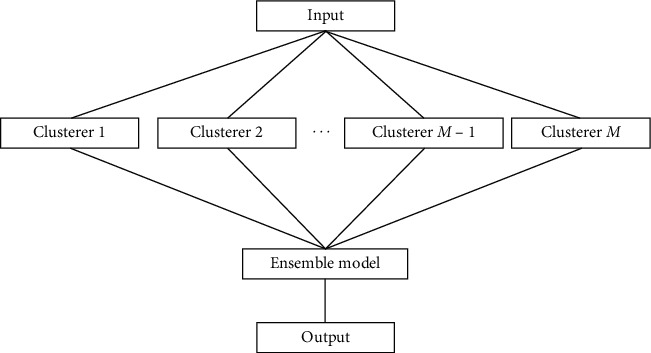
Framework of clustering ensemble model.

Ensemble learning uses multiple learners to figure out a single question, and the answer to the question is given by combining all the outputs of the learners, which can improve the result [17]. When introduced to the clustering algorithm, ensemble learning can reduce the effect caused by noise, leading to higher robustness. The clustering ensemble is usually divided into two parts, which are cluster generation and cluster fusion. Specifically, there are *n* samples in the input space *X*, learned by *M* clusterers, and a fusion algorithm combines all the clusterers to get the final learner *P* (see in Figure 1).

**Figure 2:**
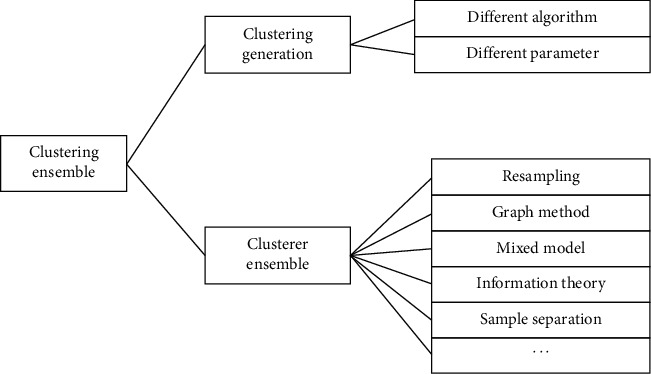
Clustering ensemble algorithm classification.

Therefore, clustering ensemble algorithms can be divided into those which focus on clusterer generation and those which focus on clusterer fusion. As for clusterer generation, there are algorithms which generate different clusterers by different clustering algorithms [1], or by the same clustering algorithm with different parameters [18, 19]. Also, dataset generation methods are used to generate different dataset from the original dataset to train different clusterers [20–22]. As for clusterer fusion, there is also plenty of research. An algorithm resamples different samples into the same cluster [23]; another one uses graph segmentation algorithm to solve clustering ensemble task [24]; furthermore, a mixed model is proposed [25]; Dempster–Shafer evidence theory is also introduced to clustering ensemble area [26]. Apart from all these, some researchers classify the sample into a transition sample and core sample and then use the core sample as the cluster segmentation basis [27, 28] (see Figure 2). Besides these two aspects, prior knowledge is also applied to improve the clustering ensemble result [29, 30].

Most research about the clustering ensemble introduces a new algorithm into the ensemble step to correct the result of certain subclusterers and combine results of all clusterers. According to research into using self-organizing map quantization error to indicate the single-pixel change in large random patterns [31], the self-organizing map cannot detect the small shift of single pixel. However, this study indicates that the self-organizing map itself can ignore a small shift in high-dimensional data, and such characteristic makes the self-organizing map able to be introduced to the ensemble step.

## 2. Materials and Methods

The SOM network can create a map between high-dimensional data and low-dimensional data, with original topology structure reserved. As a nonlinear dimension reduction algorithm, SOM trains competition layer neurons which represent prototypes of pattern in high-dimensional space to correspond with the topology structure of the original data.

**Figure 3:**
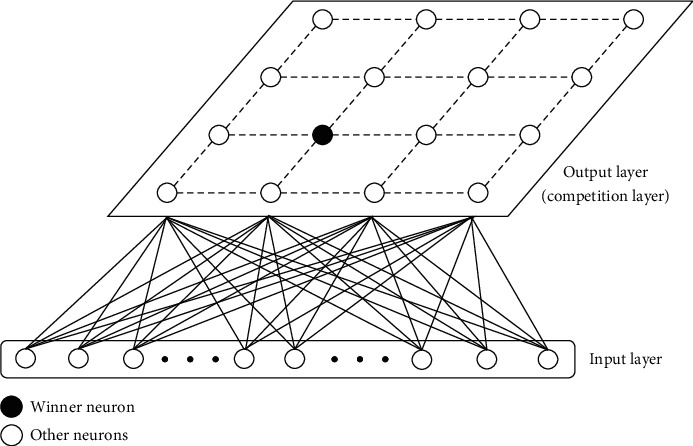
Structure of SOM network.

The network structure of SOM is shown in Figure 3. The network consists of *k* neurons where *V*={*v*_1_ … *v*_*k*_}, and each neuron has a weight vector *e*_*v*_ ∈ *R*^*d*^ whose dimension equals that of input space. Two values need to be calculated when SOM is being trained. The first is *N*(*v*_*i*_, *v*_*j*_), which is the topology distance of two neurons on the competitive layer; the second is *D*(*x*, *e*_*v*_)=*x* − *e*_*v*2_, the Euclidean distance between the input vector and the weight vector. During the training period, the target is to reduce the Euclidean distance of the weight vectors belonging to neurons that have small topology distance.

When the SOM is trained on dataset *D*, for each input *x*_*i*_ there will be a winner neuron *v* whose weight vector's Euclidean distance from the input vector is the smallest in the competition layer. Then, the weight vectors of both the winner neuron and its neighboring neurons are updated as shown in Algorithm 1, where *η* is the learning rate and *f*(·) is a neighborhood function which decreases by the increasing of *N*(*u*, *v*_*j*_).

Each round of training will force the weight vectors of the winner neuron and its neighboring neurons to move towards the input vector *x*_*i*_. Along with the iteration time *t* growing, the value of *f*(·) decreases, leading to the separation of the neurons in the competition layer. Finally, the neurons will be spontaneously gathered around different clusters. Since one or several clusters can represent a pattern in the input dataset, the Euclidean distance of weight vectors can represent the distance between different patterns, i.e., reserve the data topology structure.

**Figure 4:**
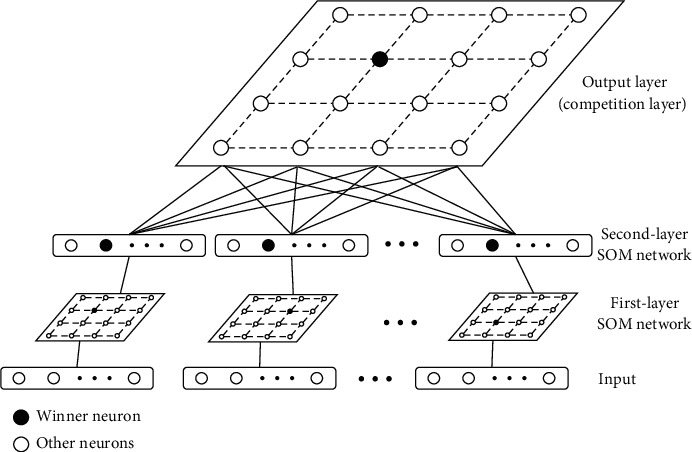
Structure of 2-layer cascaded SOM network.

Cascaded SOM combines the output of multiple competition layers as the input of another SOM model, leading to the ensemble of multiple SOM models, which can improve the performance of the learning model.  Figure 4 shows the structure of cascaded SOM (2-layer cascaded SOM as an example).

Since the competition layer of the SOM network indicates the topology structure of input patterns, the cascaded SOM needs to retain this characteristic, which cannot be provided by the original output of the SOM network. Inspired by the winner neuron of the same input pattern appearing in the same area, in order to reach the target, the output of the competition layer is changed into a one-hot vector to send the location of the winner neuron to the next layer. After the processing, the output data can be used as the training sample of the next layer, whose training step is exactly the same as shown in Algorithm 1.

The conversion of the output of the competition layer increases the dimension of the input space of the next layer, and the change of the winner of a single competition layer only changes the value on two vectors. When calculating Euclidean distance, the result would only be slightly influenced by the difference in several dimensions, which improves the robustness of the model.

## 3. Results and Discussion

**Figure 5:**
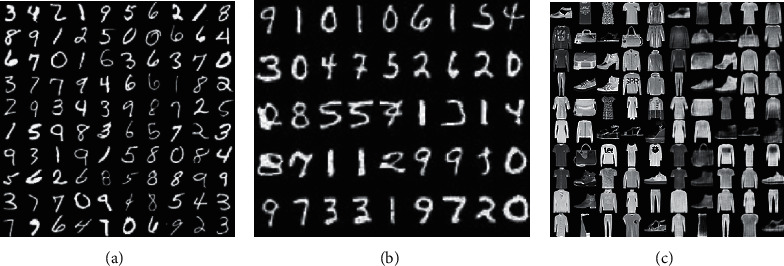
Sample display of the dataset: (a) MNIST; (b) USPS; (c) Fashion MNIST.

To measure the improvement brought by the proposed cascaded SOM to the classic SOM, several experiments are conducted on MNIST handwritten digit dataset, USPS handwritten digit dataset, and Fashion MNIST dataset (Figure 5). Data in MNIST and Fashion MNIST are 2-dimensional arrays with 28 ^*∗*^ 28 elements, while those in USPS are 2-dimensional arrays with 16 ^*∗*^ 16 elements. In the experiments, the 2-dimensional array is transferred to 1-dimensional array, which means that the array is flattened. The data in the MNIST and the Fashion MNIST are transferred into 1 ^*∗*^ 784 arrays, and the data in USPS are transferred into 1 ^*∗*^ 256 arrays. The rest of this section demonstrates the design of these experiments and discusses the results of the experiments.

In the experiments, a two-layer cascaded SOM network is used. Iteration time of training, network number of the first layer, the difference between training samples, and the network size are chosen to test their influence on the performance of the proposed algorithm. The accuracy of the clustering is utilized to evaluate the result of the clustering:

(1) $$\begin{array}{c} \text{accuracy}\left(\Omega ,C\right)=\frac{\sum_{i=1}^{N}\delta \left(c_{i},\text{map}\left(\omega_{i}\right)\right)}{N}, \end{array}$$

where N is the total number of the samples, Ω={*ω*_*i*_*|i*=1,2,…, *k*} is the set of the clusters, *C*={*c*_*j*_*|j*=1,2,…, *J*} is the real label of the sample, and  map(*ω*_*i*_) is the category of *ω*_*i*_; *δ*(*c*_*i*_, map(*ω*_*i*_)) compares *c*_*i*_ and map(*ω*_*i*_) and then outputs 1 if they are the same and 0 if they are not.

**Figure 6:**
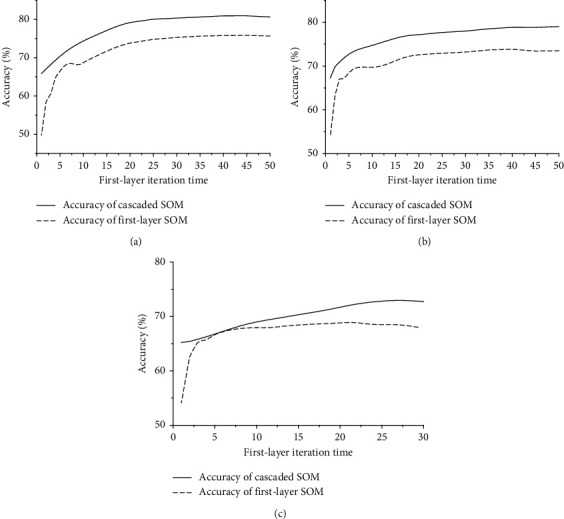
Relationship between first-layer iteration time and accuracy: (a) MNIST; (b) USPS; (c) Fashion MNIST.

When keeping the iteration time of the second layer and the network number of the first layer fixed, the accuracy of the first-layer SOM networks as well as the cascaded SOM network and the iteration time of the first-layer SOM networks are positively correlated before the first-layer SOM networks are converged (Figure 6). After that, the accuracy of the first-layer SOM networks has no obvious improvement. Compared with the first layer, the accuracy of the cascaded SOM network has a stable improvement (5% on MNIST and USPS, 4% on Fashion MNIST).

When the parameters of the first-layer network are fixed while the iteration time of the second-layer SOM network increases, the improvement of the accuracy is shown in Figure 6. As a result of the incomplete training, the accuracy has not been improved but has dropped sharply when the iteration time is only one. After several rounds of training, the accuracy improvement of the cascaded SOM network tends to a fixed value (5.7% on MNIST, 5% on USPS, 4.5% on Fashion MNIST).

The core of cascaded SOM is the exploitation of the insensitivity to changes in the values of a small number of dimensions. Therefore, the ratio of numbers of the value-changed dimension and the total dimension can influence the performance of the cascaded SOM network. The number of the first-layer networks is utilized to control the ratio mentioned above. Also, the first-layer networks should be different from each other to avoid misclassification towards the same input, which can be ensured using different initialized parameters or different training samples.

**Figure 7:**
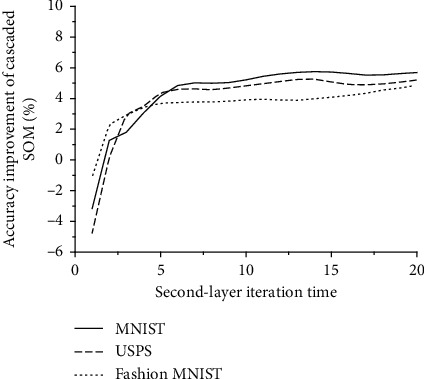
Relationship between second-layer iteration time and accuracy improvement.

When all the first-layer SOM networks are trained with the same training dataset, and the difference of the parameter is provided by randomly initialized weight vector, as shown in Figure 7, the improvement of the accuracy reaches a limit if the number of the first-layer networks increases to 13.

**Figure 8:**
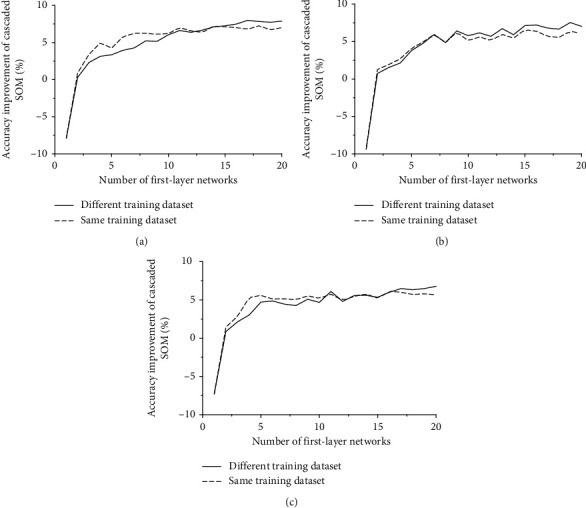
Relationship between first-layer network number and accuracy improvement: (a) MNIST; (b) USPS; (c) Fashion MNIST.

While the difference among first-layer networks is provided by both different training datasets and randomly initialized weight vector, the extra difference leads to better generalization ability. As a result, the upper limit of the accuracy is higher than the cascaded SOM network with the first-layer SOM network trained with the same dataset. However, when the number of the first-layer SOM networks is small, the generalization ability is not high enough to reduce the accuracy brought by the first-layer training dataset being different from the second-layer training dataset. Therefore, as shown in Figure 8, the cascaded SOM network with the first-layer SOM network trained with the same dataset has higher accuracy than that with first-layer SOM network trained with the different datasets when the number of first-layer SOM networks is small.

Meanwhile, the size of the first-layer SOM network can influence the performance of the SOM network. The next experiment is to figure out the effect of the size of the first-layer SOM network on accuracy. In one round of experiments, all the first-layer and second-layer SOM networks have the same size, the shape of those SOM networks is square, and the number of first-layer SOM networks is 10.

**Figure 9:**
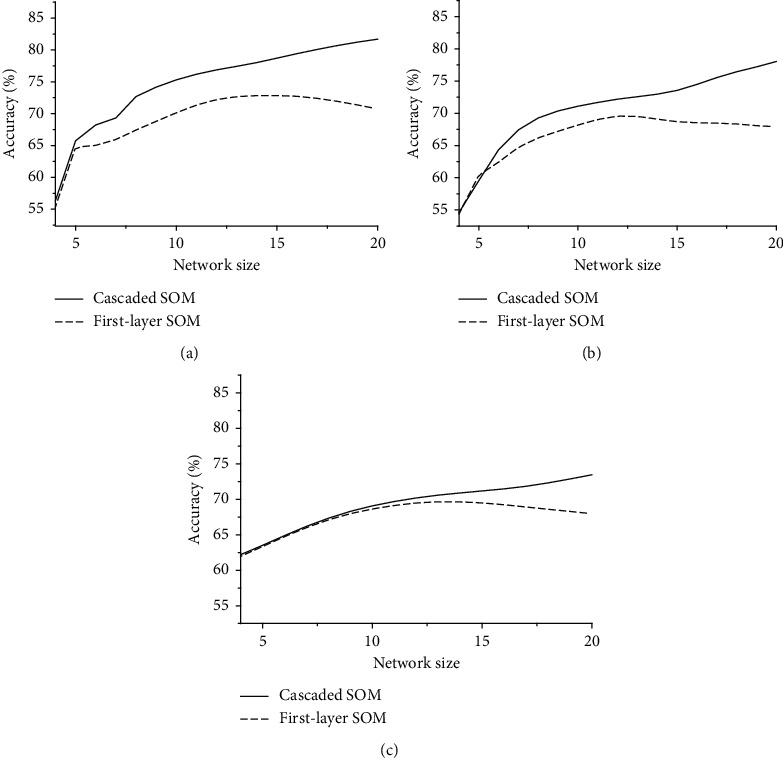
Relationship between network size and accuracy: (a) MNIST; (b) USPS; (c) Fashion MNIST.

A bigger SOM network leads to a better performance of the cascaded SOM network (Figure 9). The reason is that a bigger SOM network is beneficial to separate different clusters and reduce the number of the intercluster neurons, which is hard to judge which cluster it belongs to. This benefit can work on both the first layer and the second layer, leading to an increasing accuracy of both first-layer SOM network and cascaded SOM network. Contrary to the performance of the cascaded SOM network, there is a decrease in the accuracy of the first-layer SOM network when the size of the network keeps increasing. This phenomenon appears because each neuron can gain less knowledge if the network size keeps increasing, which raises the noise-knowledge ratio and reduces accuracy. Nevertheless, when it comes to the cascaded SOM network, larger network size means higher-dimension input of the second-layer SOM network, which improves the antinoise ability of the network, making the accuracy of the cascaded SOM network keep improving instead.

Table 1 shows the performance of the cascaded SOM network with 3 different parameter settings on MNIST, USPS, and Fashion MNIST dataset. The accuracy of the cascaded SOM is positively correlated with the iteration time of the first-layer SOM network, the iteration time of the second-layer SOM network, the number of the first-layer SOM networks, and the size of SOM network. Due to the difference among the data distribution and the data complexity of different datasets, the performance of the cascaded SOM network is slightly different, but, in general, the cascaded SOM network has better performance than the classic SOM network.

All the experiments above use the 2-layer structure.  Table 2 shows the performance of the 3-layer cascaded SOM network. The additional third competition layer can indeed provide very limited promotion to the accuracy based on the 2-layer cascaded SOM network. This indicates that the additional competition layer cannot deal with the lack of difference in the second-layer SOM network trained based on the same first-layer SOM network. Such a problem can be solved by increasing the difference between both first-layer and second-layer SOM networks, but the extra cost of training time should also be considered.

The experiments on MNIST, USPS, and Fashion MNIST indicate that the proposed cascaded SOM network can stably improve the performance of the SOM network. At the same time, like any other ensemble model, the difference among sublearners raises the generalization ability, which can result from randomly initialized weight vector or different training datasets. Though there is evidence that adding an extra layer can further improve the performance of the cascaded SOM network, the extra time cost must be considered.

The cascaded SOM network proved to be an effective algorithm, compared to ensemble classic SOM network, in improving the model. The rest of this section will compare the proposed algorithm and other clustering ensemble algorithms.

In the following experiment, ARI (adjusted Rand index) and NMI (normalized mutual information) are utilized as the evaluation indices of the clustering ensemble algorithm, which are defined as follows:

(2) $$\begin{array}{c} \text{ARI}=\frac{\sum_{i=1}^{k}\sum_{j=1}^{k}\left(n_{ij}/2\right)-\left[\sum_{i=1}^{k}\left(n_{i}/2\right)\sum_{j=1}^{k}\left(n_{i}/2\right)\right]/\left(n/2\right)}{1/2\left[\sum_{i=1}^{k}\left(n_{i}/2\right)+\sum_{j=1}^{k}\left(n_{i}/2\right)\right]-\left[\sum_{i=1}^{k}\left(n_{i}/2\right)\sum_{j=1}^{k}\left(n_{i}/2\right)\right]/\left(n/2\right)},\\ \text{NMI}\left(P^{*},P^{t}\right)=\frac{\sum_{i=1}^{k^{*}}\sum_{j=1}^{k^{t}}n_{j}\log\left(n.n_{ij}/n_{i}.n_{j}\right)}{\sqrt{\left(\sum_{i=1}^{k^{*}}n_{i}\log\left(n_{i}/n\right)\right)\left(\sum_{j=1}^{k^{t}}n_{j}\log\left(n_{j}/n\right)\right)}}, \end{array}$$

where *n* is the total number of samples, *n*_*ij*_ is the number of objects in the intersection of clusters *c*_*i*_ ∈ *P*^*t*^ and *c*_*j*_ ∈ *P*^*∗*^, *P*^*∗*^ is the clustering result and *P*^*t*^ is the true class numbers of the samples, *n*_i_ and *n*_j_ are the numbers of samples in clusters *c*_*i*_ ∈ *P*^*t*^ and *c*_*j*_ ∈ *P*^*∗*^, respectively, and (*n*/2) is the binomial coefficient (*n*!)/2!(*n* − 2). The maximum value of ARI is equal to 1. The maximum value of NMI is equal to 1.

Eight real-world datasets from the UCI Machine Learning Repository were used, including Iris, Wine, Thyroid, Multiple Features (Mfeatures), and Ionosphere dataset.  Table 3 shows the details of these datasets. BCW has an attribute with missing values in some objects, which is removed. The second attribute in the Ionosphere dataset is also removed.

Six other clustering ensemble algorithms are chosen, which are CO-Average (Co-Association method using the average linkage method) [32], ONCE-Average (Object-Neighborhood Clustering Ensemble using the average linkage method) [33], DSCE (Dual-Similarity Clustering Ensemble method) [34], ACE (Adaptive Clustering Ensemble) [35], DICLENS (Division Clustering Ensemble) [36], and MCLA (Meta-Clustering Algorithm) [1].

The results of the experiments are shown in Tables 4 and 5. The proposed algorithm has the best performance on several datasets (Wine, Mfeatures, and Glass) and has a relatively good performance on the other datasets too.

## 4. Conclusions

In this paper, a new clustering ensemble approach is investigated. The proposed method is based on introducing a cascaded structure to the self-organizing map. To test the clustering ensemble ability of the algorithm, experiments are conducted on MNIST, USPS, and Fashion MNIST dataset. The experiments on those datasets indicate that the proposed cascaded SOM network has the following characteristics:

1. It can stably improve the performance of the SOM network.
2. The difference among sublearners, which can result from randomly initialized weight vector or different training datasets, raises the generalization ability.
3. The 2-layer cascaded SOM network can improve the performance of the SOM network, while the 3-layer cascaded SOM network can only slightly increase the accuracy compared to that of a 2-layer one, yet the extra time cost must be considered.

The proposed algorithm is proved to have advantages on several datasets, compared with other clustering ensemble algorithms.

Also, the proposed algorithm can still be improved. For now, all the methods, except using different training datasets, to improve the performance of the cascaded SOM are increasing the data dimension, which increases the training time cost at the same time. Therefore, the network can hardly reach its theoretical limit in practical application due to time cost control.

## Figures and Tables

**Algorithm 1:**
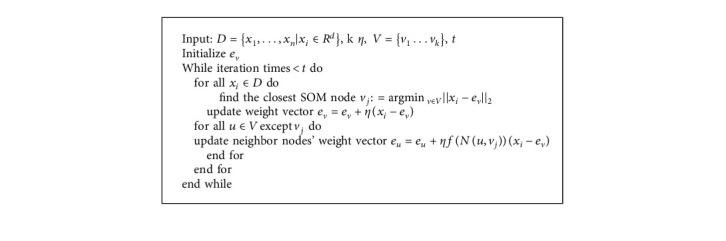
Learning rule of SOM network.

**Table 1:** Accuracy improvement of cascaded SOM network.

Network parameter	MNIST	USPS	Fashion MNIST (%)
Parameter setting A	2.68	3.89	−0.51
Parameter setting B	5.83	6.23	1.43
Parameter setting C	10.51	11.05	4.63

A: iteration number of the first layer: 30; iteration number of the second layer: 30; number of the first-layer networks: 10; network size: 10 × 10. B: iteration number of the first layer: 50; iteration number of the second layer: 50; number of the first-layer networks: 10; network size: 15 × 15. C: iteration number of the first layer: 50; iteration number of the second layer: 50; number of the first-layer networks: 20; network size: 20 × 20.

**Table 2:** Accuracy of each layer in 3-layer cascaded SOM network.

Network size	Layer	MNIST (%)	USPS (%)	Fashion MNIST (%)
10 × 10	1st	73.41	76.38	70.32
	2nd	80.02	81.73	72.19
	3rd	80.74	82.25	72.45
20 × 20	1st	73.68	76.18	70.37
	2nd	81.27	82.42	73.37
	3rd	81.7	83.14	73.66

**Table 3:** Details of the datasets used in experiments.

Dataset	Sample number	Feature number	Cluster number
Iris	150	4	3
Wine	178	13	3
Thyroid	215	5	3
Mfeatures	2000	2	10
Glass	214	9	6
BCW	683	9	2
Soybean	47	35	4
Ionosphere	351	34	2

**Table 4:** The average performance of ten runs for each dataset measured by ARI.

	CO-Average	ONCE-Average	DSCE	ACE	DICLENS	MCLA	Cascaded SOM
Iris	0.725	0.726	0.732	**0.734**	0.680	0.723	0.725
Wine	0.369	0.369	0.377	0.371	0.369	0.372	**0.380**
Thyroid	0.559	0.584	0.609	**0.613**	0.582	0.563	0.592
Mfeatures	0.315	**0.316**	**0.316**	0.314	0.290	0.308	**0.316**
Glass	0.509	0.526	0.528	0.535	0.392	0.534	**0.536**
BCW	**0.849**	0.847	**0.849**	**0.849**	0.842	**0.849**	0.848
Soybean	0.547	0.550	0.578	0.532	**0.632**	0.548	0.572
Ionosphere	0.163	0.166	**0.169**	0.165	0.161	0.166	0.166

The bold values mean the best ARI value of the different algorithms for each dataset.

**Table 5:** The average performance of ten runs for each dataset measured by NMI.

	CO-Average	ONCE-Average	DSCE	ACE	DICLENS	MCLA	Cascaded SOM
Iris	0.751	0.752	0.763	**0.766**	0.757	0.749	0.757
Wine	0.428	0.428	0.432	0.429	0.427	0.429	**0.434**
Thyroid	0.434	0.473	0.480	**0.531**	0.501	0.418	0.492
Mfeatures	0.479	0.479	0.479	0.478	0.468	0.475	**0.480**
Glass	0.712	0.725	0.725	0.726	0.617	0.728	**0.731**
BCW	0.750	0.749	0.750	**0.751**	0.742	**0.751**	0.748
Soybean	0.717	0.723	0.756	0.712	**0.822**	0.717	0.785
Ionosphere	0.122	0.124	**0.128**	0.123	0.119	0.124	0.126

The bold values mean the best NMI value of the different algorithms for each dataset.

## Data Availability

The data used to support the findings of this study are included in the article.
